# Developing benthic monitoring programmes to support precise and representative status assessments: a case study from the Baltic Sea

**DOI:** 10.1007/s10661-020-08764-7

**Published:** 2020-11-27

**Authors:** Henrik Nygård, Mats Lindegarth, Alexander Darr, Grete E. Dinesen, Ole R. Eigaard, Inga Lips

**Affiliations:** 1 Marine Research Centre, Finnish Environment Institute, Helsinki, Finland; 2 Department of Marine Science–Tjärnö, University of Gothenburg, Gothenburg, Sweden; 3 Leibniz Institute for Baltic Sea Research Warnemünde, Rostock, Germany; 4 National Institute of Aquatic Resources, Technical University of Denmark, Kgs. Lyngby, Denmark; 5 Department of Marine Systems, Tallinn University of Technology, Tallinn, Estonia

**Keywords:** Benthic habitats, Monitoring design, Marine management, Marine Strategy Framework Directive, Uncertainty, Baltic Sea

## Abstract

Benthic habitats and communities are key components of the marine ecosystem. Securing their functioning is a central aim in marine environmental management, where monitoring data provide the base for assessing the state of marine ecosystems. In the Baltic Sea, a > 50-year-long tradition of zoobenthic monitoring exists. However, the monitoring programmes were designed prior to the current policies, primarily to detect long-term trends at basin-scale and are thus not optimal to fulfil recent requirements such as area-based periodic status assessments. Here, we review the current monitoring programmes and assess the precision and representativity of the monitoring data in status assessments to identify routes for improvement. At present, the monitoring is focused on soft-bottoms, not accounting for all habitat types occurring in the Baltic Sea. Evaluating the sources of variance in the assessment data revealed that the component accounting for variability among stations forms the largest proportion of the uncertainty. Furthermore, it is shown that the precision of the status estimates can be improved, with the current number of samples. Reducing sampling effort per station, but sampling more stations, is the best option to improve precision in status assessments. Furthermore, by allocating the sampling stations more evenly in the sub-basins, a better representativity of the area can be achieved. However, emphasis on securing the long-term data series is needed if changes to the monitoring programmes are planned.

## Introduction

The seafloor provides a wide range of habitats inhabited by benthic communities and delivering a diverse set of ecosystem services (Galparsoro et al. 2014). The functioning of these habitats, e.g. in carbon and nutrient cycling and retention or as feeding and nursery grounds for higher trophic levels (Kritzer et al. 2016; Griffiths et al. 2017) is, however, threatened due to increasing pressure from anthropogenic activities in the marine environment (Halpern et al. 2015). Reliable estimates of the environment’s condition set the base for successful management of human activities to secure a sustainable provision of ecosystem services. In Europe, management of the marine areas is guided by environmental policies, such as the European Union’s Marine Strategy Framework Directive (MSFD: EU 2008, 2017), Water Framework Directive (WFD; EU 2000) and Habitats Directive (HD; EU 1992), all requiring periodic environmental status assessments including the benthic component. The MSFD utilises WFD and HD assessments where relevant, but also requires assessments of each broad habitat type with respect to the area lost and the area adversely affected by human activities (EU 2017), implying an increased focus on spatial aspects. Furthermore, at a regional scale, the Baltic Sea Action Plan (BSAP; HELCOM 2007) outlines the environmental goals in the Baltic Sea.

Benthic communities are proficient indicators of environmental changes, as changes in the community composition reflect changes in the environment (Leppäkoski 1975; Pearson and Rosenberg 1978; Dauer 1993). To detect changes in benthic communities and provide reliable status assessments at regional scale, appropriate and well-designed monitoring programmes are needed (Van Hoey et al. 2019). In the Baltic Sea, there is a >-50-year-long tradition of monitoring soft-bottom macrofauna communities, providing unique time series to study changes over time. However, the emerging paradigm of integrated status assessments at aggregated spatial and temporal scales as promoted by the MSFD and the WFD puts new demands on the design of monitoring programmes.

A monitoring programme allowing for precise status assessments requires that samples are representatively allocated in time and space, and thus, the sources of variability in the monitored parameter need to be quantified. Estimating the temporal and spatial components of this variability from large datasets and incorporating them into overall estimates of uncertainty can help achieving reliable status assessments. Standardised statistical procedures (e.g. Carstensen and Lindegarth 2016) can be applied to estimate assessment precision and involves identification of the dimensioning of the monitoring programme (i.e. the number of years, stations, and samples per assessment unit), estimation of variance components, and assessment of estimation error using formulae for error propagation. These analytical tools can also be used in subsequent evaluation of alternative scenarios for monitoring.

HELCOM, the Baltic Marine Environment Protection Commission, coordinates the implementation of the BSAP and the regional implementation of MSFD in the Baltic Sea. It has established common monitoring guidelines and common core indicators to facilitate a consistent status assessment (HELCOM 2013a, 2017). Although common guidelines exist, no regionally coordinated standard monitoring programme is in place, but the data available for regional assessments rather stem from a combination of national monitoring programmes. The scope of many national monitoring programmes is to collect long-term data series for temporal trend analyses, but although originally not always intended for it, the derived data is now also used for MSFD status assessments by EU countries. In the latest holistic assessment of the Baltic Sea (HELCOM 2018a, b), the open-sea benthic habitats were assessed primarily using the biodiversity core indicator “State of the soft-bottom macrofauna community” (HELCOM 2018c). But up to now, it has never been tested whether the combination of this indicator with the available monitoring data fits the requirements of the new demands arising from the MSFD and, hence, whether the achieved results reliably estimate the status of the benthic habitats in the Baltic Sea.

Consequently, in this paper, we review the current open-sea benthic monitoring programmes in the Baltic Sea, with respect to the assessment requirements of current environmental policies and management needs. Specifically, we address the coverage of broad habitat types and regional consistency in monitoring methods. Furthermore, we use the monitoring data to evaluate how representative and precise assessments of environmental status the monitoring programmes can deliver with the currently available indicator. To do so, we geographically analyse the distribution of sampling stations and estimate the variance components and precision of the current indicator assessment. Based on these analyses, we then evaluate monitoring scenarios and discuss possibilities to improve and optimise the monitoring programmes to provide representative and confident periodic status assessments, while still securing following up on long-time trends.

## Material and methods

### Review of current benthic monitoring programmes

Information on current benthic monitoring programmes in the Baltic Sea was collected through a survey that was sent out to Baltic Sea monitoring experts. Information on spatial and temporal coverage, habitat type targeted, monitoring methodology and parameters, and survey design was asked for. Replies were received from all countries except Latvia and Russia. For Latvia, information available in the HELCOM Map and Data Service was used.

To evaluate the appropriateness of current monitoring programmes with respect to current policy requirements, features such as regional consistency in monitoring methodology, spatial coverage, and coverage of broad habitat types were addressed. MSFD requires an assessment of each broad habitat type at a regional scale, reflecting biogeographic differences in species composition of the broad habitat types. In the Baltic Sea, the biogeographic pattern in species distributions is defined by gradients in both salinity and climate, which are reflected in the subdivision into assessment units (HELCOM 2013b). Thus, HELCOM assessment units are a relevant scale for MSFD assessments of benthic habitats. The focus of this paper is on monitoring open-sea assessment units, and thus, the information on coastal monitoring (i.e. coastal assessment units) is not included here. The term “sub-basin” is subsequently used to refer to the open-sea assessment units. For spatial overlays with broad habitat types and for calculating the proportion of broad habitat types in the sub-basins, information from EUSeaMap (version published 2016-09-30; Populus et al. 2017) was used. The EUSeaMap is currently the only available map of broad habitat types at a regional scale.

### Analyses of monitoring data used in assessments

To analyse the sufficiency of current monitoring programmes to enable accurate status assessments, we used the HELCOM core indicator “State of the soft-bottom macrofauna community” (HELCOM 2018c) and the data used for the HOLAS II assessment (http://metadata.helcom.fi/geonetwork/srv/eng/catalog.search#/metadata/0fdc6cb9-fa15-4ba3-9d73-8aeddb5cff64). The indicator uses the benthic quality index (BQI; Rosenberg et al. 2004; Leonardsson et al. 2009), which is based on the zoobenthic community structure: the proportion of taxa showing different sensitivity to pressures, species richness, and total abundance. In the indicator, two types of species sensitivity values are used, (i) a classification based on literature and expert knowledge into discrete categories with the sensitivity values 1, 5, 10, and 15 (from tolerant to sensitive; Leonardsson et al. 2009; later referred to as BQI1) and (ii) calculated sensitivity values on a continuous scale (~ 1–16) accounting for differences in salinity, depth, and sampling gear (Schiele et al. 2016; later referred to as BQI2). Both BQI1 and BQI2 are calculated using the BQI formula as presented in Leonardsson et al. (2009), but BQI1 tend to have higher BQI values than BQI2 as a consequence of the different method used to define species sensitivity values. Separate precision analyses were performed for the two versions of BQI. BQI1 was used in the Bothnian Bay, The Quark, Bothnian Sea, Åland Sea, Northern Baltic Proper, and Western Gotland Basin, whereas BQI2 was used in the Gulf of Finland, Gulf of Riga, Eastern Gotland Basin, Gdansk Basin, Bornholm Basin, Arkona Basin, and Bay of Mecklenburg and Kiel Bay. The station network and data used here slightly differ from the station network used in the analyses of current monitoring programmes as (i) the indicator is targeting soft-bottom infauna, (ii) national monitoring programmes have been revised since the assessment period 2011–16, and (iii) additional stations and data from research projects, not included in the current monitoring programmes, were used in HOLAS II. In the estimate of variance components, all data from HOLAS II were used to include as much variability as possible, but in the analyses of representativity and precision of the monitoring network, data from research projects were excluded, for the results to reflect the actual monitoring network.

#### Representativity

Representativity of the monitoring data in the assessment was evaluated both in temporal and spatial terms. Temporal representativity was assessed as how well the assessment period was covered, i.e. how many years were sampled. Spatial representativity in the sub-basins was assessed in terms of both number of sampling stations and the geographical distribution of stations. In general, it is desirable that the monitoring programme covers (represents) all geographic parts of the sub-basin. Here, a grid-based approach was applied to estimate the current coverage of the sub-basins. However, a drawback with this approach is that the result of such an analysis depends on the spatial resolution (grid-size). Hence, we also evaluated at what resolution the current number of stations could cover the entire sub-basin.

#### Estimation of variance components

Like any other biological, chemical, or physical variable, the BQI show temporal and spatial variability due to factors that are more or less predictable (e.g. season, depth, and habitat) or unpredictable (e.g. among stations within a sub-basin or habitat). The extent to which these factors influence the BQI can be estimated using linear models, and the contribution of different factors to the uncertainty of a monitoring design can be assessed (Carstensen and Lindegarth 2016). The effects of predictable (≈“fixed”) factors are accounted for and uncertainty due to such factors can be removed.

The linear model used to estimate important spatial and temporal components from the zoobenthic data contains a number of *fixed* and *RANDOM* components (note that the latter are denoted by capital letters, Eq. 1).

1 $$ y=\mu + bh+ sb+ YR+ bh\times sb+ ST\left( bh, sb\right)+ YR\times ST\left( bh, sb\right)+ RES $$

Because the important assessment units in the MSFD are the sub-basins (*sb*), these are considered fixed in the same way as the broad habitat (*bh*) types. The random components are years within assessment periods (*YR*), stations (*ST*) within broad habitat types and sub-basins, interactive spatiotemporal variability (*YR* × *ST*) and the unexplained residual within stations and years (*RES*). Thus, this model accounts for systematic differences among broad habitat types but estimates overall random variability within sub-basins. Estimates from this model can be used to assess uncertainty of monitoring programmes that are planned for individual habitat types. If on the other hand monitoring is planned within sub-basins without careful consideration of habitat type (as in our case where the indicator is only targeting soft-bottom infauna or if the information on habitat type is not reliable), *bh* can be removed from the model (Eq. 2) and any variability due to effects of habitat type will now be included in the *ST* component.

2 $$ y=\mu + sb+ YR+ ST(sb)+ YR\times ST(sb)+ RES $$

Variance components were estimated using maximum likelihood procedures using the library “lme4” (routine lmer, Bates et al. 2015) in R (R Core Team 2019).

#### Precision estimate

The precision of a mean estimate (i.e. how close the estimated mean is to the unknown “true” mean), is a critical aspect of the performance of a sampling program. The HELCOM COMBINE manual (HELCOM 2017) recommends a relative error of ≈ 20% of the mean as an acceptable error. Although the COMBINE manual only define the acceptable error per sampling station, the 20% relative error of the mean was in this study also used as a benchmark for years, sub-basins, and assessment period. The HOLAS II assessment addressed the “certainty of classification” (≈ “confidence in classification” according to the terminology used in Lindegarth et al. 2013), which is related to precision but is also affected by the distance to the class boundary and is therefore not entirely comparable to measures of uncertainty presented here.

The uncertainty of any mean estimate of an indicator is determined by the monitoring design and effort and the size of spatial and temporal variability. The central spatial units for status assessment in the Baltic Sea for the MSFD are the broad habitat types within the sub-basins (HELCOM assessment units), and the fundamental temporal unit is the 6-year assessment period. However, the indicator is not differentiating among broad habitat types, but only targeting soft-bottoms, i.e. where grab samples can be taken. A caveat when overlaying sampling stations with habitat maps is the differing scales at which monitoring is undertaken and the maps are modelled. Although a monitoring station ideally should represent a wider surrounding (at minimum a radius of 50 m; Leonardsson and Blomqvist 2015) than the 0.1 m^2^ that the van Veen grab samples, habitat maps (especially at regional level) often have a considerably coarser scale. For example, the EUSeaMap used in this study applies a 250-m resolution (Populus et al. 2017). Therefore, the analyses in this study focus primarily on uncertainty within sub-basins and assessment periods (i.e. using the variance components from Eq. 2). The fact that monitoring is structured in time (sampling in multiple years) and space (sampling in stations and replicates) means that several components of variability need to be accounted for in estimates of overall uncertainty.

As a general rule, in a design where sampling sites are revisited among years, the crucial components for estimating uncertainty at this scale are those among years, sites within sub-basins, interactive variability, and replicate samples (i.e. ${s}_{YR}^2$, ${s}_{ST}^2$, ${s}_{YR\times ST}^2$, and ${s}_{RES}^2$, respectively). These components are combined using formulae for error propagation into an overall sampling variability of the total mean according to:

3 $$ V\left[{\overline{y}}_{\mathrm{period},\mathrm{sub}-\mathrm{basin}}\right]=\frac{s_{YR}^2\times \left(1-\frac{a}{Y}\right)}{a}+\frac{s_{ST}^2}{b}+\frac{s_{YR\times ST}^2}{ab}+\frac{s_{RES}^2}{ab n} $$

The presented formulae for error propagation strictly assume balanced data. The analyses presented here have used averages of *a*, *b*, and *n* in the respective sub-basins, stations, and years. A more general formulation was presented in Carstensen and Lindegarth (2016), but for the purposes of this study, the simpler approach was considered sufficiently robust.

The contribution of each component to the overall uncertainty can be modulated by the number of years of sampling within a period (*a*, where *Y* is the maximum number of years *Y* = 6), the number of sites sampled within a sub-basin (*b*), and the number of replicate samples per site (*n*). The standard error of the total mean is calculated as:

4 $$ SE=\sqrt{V\left[{\overline{y}}_{\mathrm{period},\mathrm{sub}-\mathrm{basin}}\right]} $$

Additionally, for the assessment and design of monitoring programmes, it is also of interest to assess the uncertainty of estimates at the scale of individual years and stations. For example, excessive uncertainty of mean estimates at the scale of individual years will severely affect possibilities to find correlative patterns related to large-scale climatic factors and excessive uncertainty at the scale of years and stations may preclude analyses of individual time series. Thus, the overall variability within sub-basins and years (Eq. 5) and stations and years (Eq. 6) were calculated as:

5 $$ V\left[{\overline{y}}_{\mathrm{year}\times \mathrm{sub}-\mathrm{basin}}\right]=\frac{s_{ST}^2}{b}+\frac{s_{YR\times ST}^2}{b}+\frac{s_{RE\mathrm{S}}^2}{bn} $$

6 $$ V\left[{\overline{y}}_{\mathrm{year}\times \mathrm{station}}\right]=\frac{s_{RES}^2}{n} $$

#### Scenario modelling

After estimating the baseline precision of the monitoring and assessments, the expected precision of monitoring BQI under a range of scenarios at different scales was modelled. The figures produced can be used to graphically estimate the expected precision within individual sub-basins. In the modelling of different scenarios, the variance estimates derived from the model without accounting for habitats (Eq. 2) were used. The estimates of SE or CV can thus be taken as conservative (i.e. accounting for habitats would most likely reduce SE slightly). As the dimensioning at smaller spatial scales potentially influences the precision at larger scales, the modelling started at the scale of stations within years, proceeded to sub-basins within years, and ended up with sub-basins within periods, which is the most important assessment scale for status assessment.

In terms of monitoring assessment periods, it is also worth noting that there are alternative approaches for selecting stations. The traditional and most intuitive approach for assessing time series is to revisit the same stations year after year. This is the so-called crossed or orthogonal sampling design that allows for estimation of changes from year to year within individual stations. The extreme of another alternative approach is to randomly select new stations each year of sampling. This is called a “nested” sampling design and it means the sampled stations are unique for each year. The benefit of such a design is that a larger number of stations can be sampled within the assessment period, but on the other hand, the change within each individual station among years cannot be evaluated. Nevertheless, considering the strong impact of number of stations on the precision, this design may potentially be more efficient for status assessment. The sampling variability for periods using the nested design is shown in Eq. 7 (note that ${s}_{ST(YR)}^2$ can be estimated as ${s}_{ST}^2+{s}_{YR\times ST}^2$).${s}_{ST(YR)}^2$ can be estimated as ${s}_{ST}^2+{s}_{YR\times ST}^2$).

7 $$ V\left[{\overline{y}}_{\mathrm{period},\mathrm{sub}-\mathrm{basin},\mathrm{nested}}\right]=\frac{s_{YR}^2\times \left(1-\frac{a}{Y}\right)}{a}+\frac{s_{ST(YR)}^2}{ab}+\frac{s_{RES}^2}{ab n} $$

## Results

### Review of current monitoring

Benthic monitoring is carried out in the open areas of the Baltic Sea by all bordering countries (no information available from Russia). The oldest monitoring programmes started in the 1960s, with the most recent starting in the 1990s. The national monitoring programmes are mainly carried out in the sea areas closest to the respective country and no country has a pan-Baltic sampling network. The regional Baltic Sea monitoring is thus a combination of the national monitoring programmes. Given that most of the monitoring is on soft-bottoms, macrofauna and especially infauna are the focus of the monitoring programmes. Three countries (Denmark, Germany, and Poland) reported monitoring of angiosperms or macroalgae in the open sea. Only two countries (Denmark and Germany) reported monitoring especially designed for the Habitats Directive.

In the open sea, all monitoring programmes use fixed stations, although there have been some changes in the station network over the years. Some new types of sampling designs have also been introduced lately, e.g. (1) a cluster design where single stations with several replicates samples have been exchanged to a cluster of several relatively closely situated stations with only one sample per station (Sweden), and (2) depth transects where stations are placed at different depths to better understand the community changes due to the varying depth of the hypoxic water layer in open-sea deep basins (Estonia).

The HELCOM COMBINE monitoring guidelines form the basis of most national monitoring programmes, but differences in sampling methodology still exist. Zoobenthic sampling is most often done using van Veen grabs, but one country uses HAPS corer. Samples are usually collected in spring/early summer (April–June; 7 countries), but sampling in autumn (October–November) is also done by one country. The sampling frequency is usually once per year, although in some cases, it has lately been reduced to once every second year. Samples are sieved using either 1-mm (5 countries), 0.5-mm (2 countries), or 0.25-mm (1 country) sieve mesh sizes. For preservation of samples, formalin is the most common medium (5 countries), but also, ethanol (2 countries) and deep freezing (1 country) of samples are used. Species richness, abundance, and biomass are recorded in all monitoring programmes, and some countries also record size distribution of bivalves (3 countries), crustaceans (1 country), and polychaetes (1 country). The data is stored in national databases and in most cases reported to the COMBINE database hosted at ICES (https://ocean.ices.dk/helcom/).

Monitoring programmes dedicated to habitats and HD requirements are primarily aimed at sand banks and reefs and target both fauna and vegetation. These monitoring programmes have a more extensive suite of sampling methods including underwater video transects and frames, as well as dredges. No regional guidelines for these monitoring methods are yet in place.

#### Monitoring station network vs sub-basins and habitat types

**Fig. 1:**
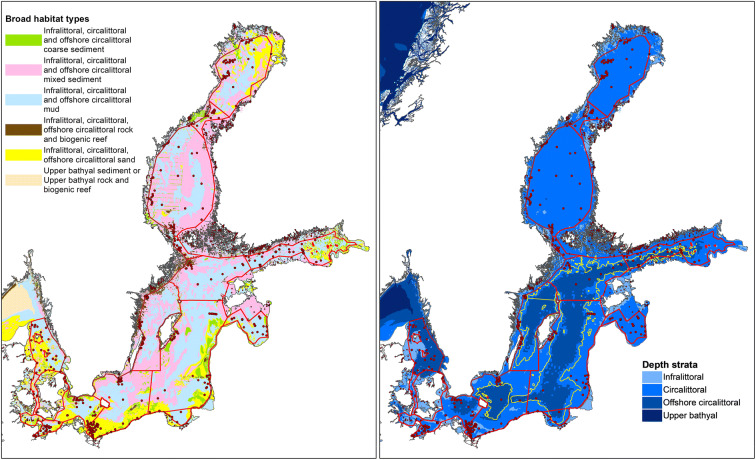
Distribution of sediment substrate types (left) and depth strata (right) in the Baltic Sea, based on EUSeaMap (version published 2016-09-30; Populus et al. 2017). The red lines outline the HELCOM open-sea assessment units. Zoobenthic monitoring stations are indicated with red dots. The striped yellow line (map on right) outlines the 60-m-depth isoline, which was used in HOLAS II as a proxy for the permanent halocline

**Fig. 2:**
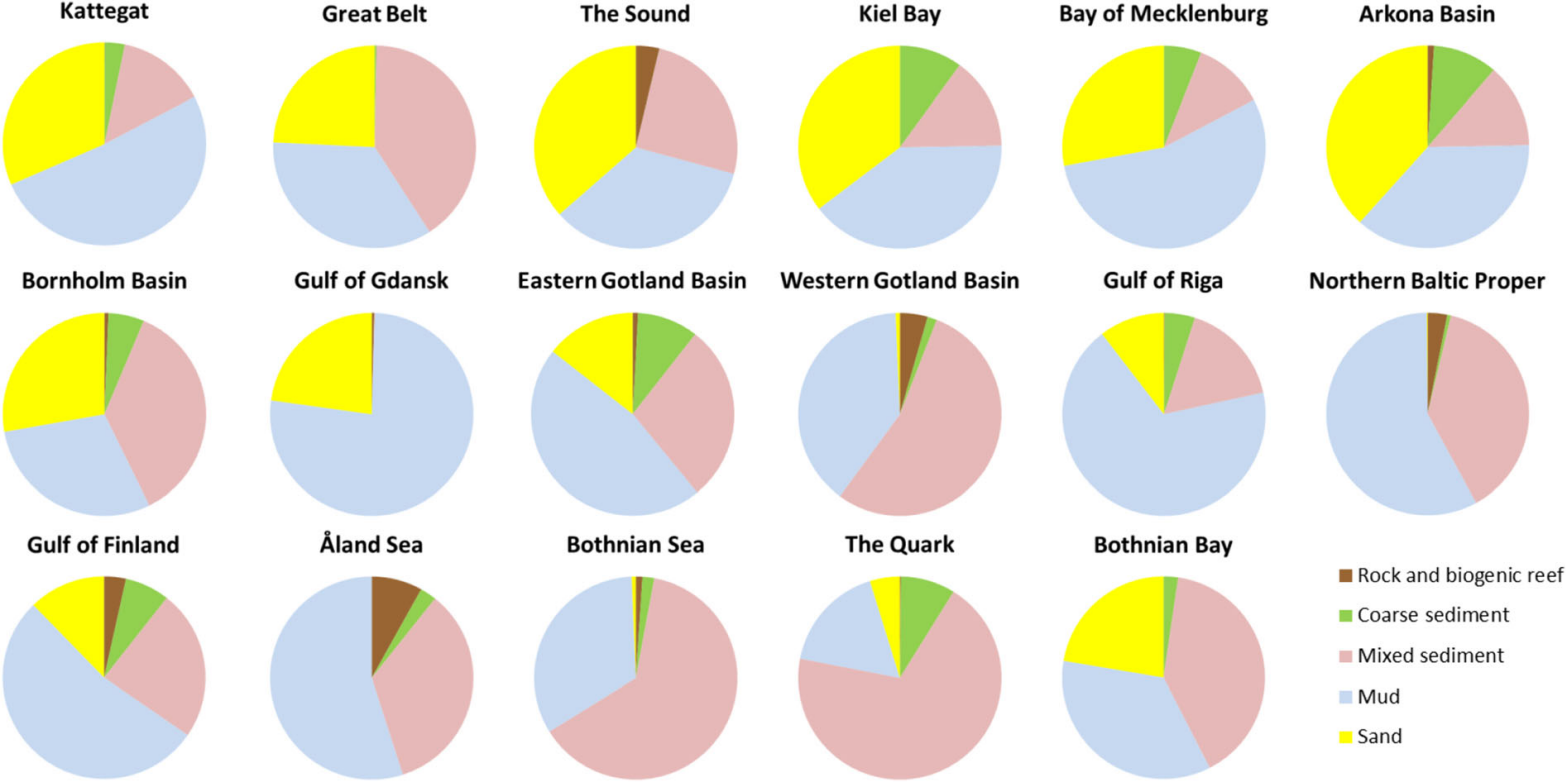
Distribution of substrate types in the HELCOM open-sea spatial assessment units based on EUSeaMap (Populus et al. 2017)

The current Baltic Sea benthic monitoring station network covers all sub-basins (Fig. 1). Dividing the stations among the broad habitat types shows that most stations are found in the circalittoral depth stratum and on mud or mixed sediments, and within sub-basins, the dominating broad habitat types generally have most stations (Table 1). In the southwestern Baltic Sea, monitoring on sand and coarse sediments is also common. In the open-sea areas, the main substrate types were mixed, mud, and sand. These three substrate types covered > 88% of the open-sea areas in all sub-basins (Fig. 2). Infralittoral areas are common west of the Arkona Basin, but in the rest of the Baltic Sea, the open-sea circalittoral and offshore circalittoral depth strata cover > 90%.

**Table 1:**
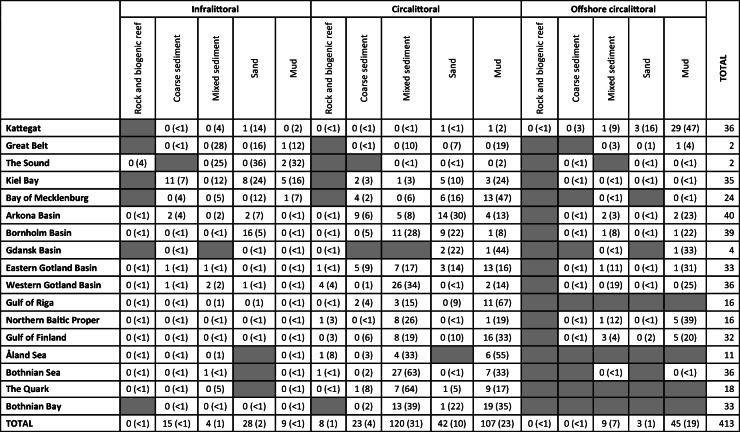
Number of monitoring stations in Baltic Sea sub-basins and MSFD broad habitat types. The number in brackets shows the percentage of sub-basin area covered by the broad habitat type. The broad habitat types are based on EUSeaMap. Grey cells indicate that the broad habitat type does not occur in the sub-basin

### Analyses of monitoring data from soft-bottoms used in assessments

#### Representativity

In the HOLAS II assessment period 2011–2016, approximately 1750 zoobenthic samples from 300 unique monitoring stations were available (Table 2). The number of samples varied among sub-basins and years, but approximately 300 samples were available per year. Similarly, the number of stations varied among units and years, but in general, there were roughly 150 stations available per year.

**Table 2:** The number of years sampled and range of samples and stations among years in different sub-basins

Sub-basin	Years sampled	Minimum samples	Maximum samples	Minimum stations	Maximum stations	Unique stations
Kiel Bay	1	0	2	0	2	2
Bay of Mecklenburg	6	21	61	5	15	16
Arkona Basin	6	13	23	11	13	17
Bornholm Basin	6	25	56	11	22	25
Gdansk Basin	1	0	15	0	3	3
Eastern Gotland Basin	6	19	61	11	34	35
Western Gotland Basin	6	5	21	5	21	23
Gulf of Riga	6	12	34	4	14	15
Northern Baltic Proper	6	6	9	6	9	17
Gulf of Finland	6	8	45	3	23	28
Åland Sea	5	0	13	0	11	11
Bothnian Sea	6	36	63	20	35	35
The Quark	6	6	27	3	16	18
Bothnian Bay	6	22	55	14	37	53

Overall, the temporal coverage in the different areas appears to be good, except for the Kiel Bay and the Gdansk Basin, which only had samples from 1 year (Table 2). Another aspect of temporal representativity is how much the spatial coverage varies among years. In particular, a smaller number of stations in 1 year may lead to biased means for that particular year. From this perspective, the Bothnian Sea and Bothnian Bay are the best-sampled ones followed by the Eastern Gotland Basin, Bornholm Basin, and Arkona Basin.

In terms of geographic representativity, assessments are a little more complicated. The analyses show that the density of stations in the monitoring programmes ranges from 4.5 to 0.6 stations per 1000 km^2^ for the individual sub-basins (3.8 for the whole Baltic, Table 3). Theoretically, densities > 1 station per 1000 km^2^ appear to be sufficient to cover ≈ 100% of the cells at a resolution of 20–40-km grids. This includes both relatively large sub-basins (e.g. Bothnian Bay and the Western Gotland basin), as well as smaller ones (e.g. The Quark and Åland Sea), with Gulf of Riga being an exception. But, using the current spatial distribution, the coverage in the basins with such station densities ranges from 14 to 53% at 20–40-km grids. As expected, many of the larger sub-basins have a low density of stations, and with the current effort, full coverage can only be achieved at a lower resolution. The current design achieves a cover of 13–73% at the resolution where full coverage could be achieved with the current number of stations (Table 3).

**Table 3:** Number of unique stations, area of the sub-basin, and station density in the sub-basins. For the different grid resolutions used in this study, the number of cells per sub-basin and the percentage of cells covered (at least one station) with current monitoring are shown. Number of cells per grid resolution includes also cells only partially covering the sub-basin. Cells in italics indicate the resolution that could potentially be covered using the current number of stations

Sub-basin	Number of stations	Area (km^2^)	Stations per 10^3^ km^2^	Number of cells per grid resolution				Percentage of cells with monitoring stations per grid resolution			
				10 km	20 km	40 km	80 km	10 km	20 km	40 km	80 km
Kiel Bay	2	3450	0.58	43	17	8	4	5	12	25	50
Bay of Mecklenburg	16	4475	3.58	54	*17*	7	4	26	53	57	50
Arkona Basin	17	15,300	1.11	171	53	*19*	7	8	15	21	57
Bornholm Basin	25	41,750	0.60	441	127	37	*15*	4	11	27	47
Gdansk Basin	3	4575	0.66	56	20	8	4	5	15	38	50
Eastern Gotland Basin	35	40,000	0.88	472	151	58	*23*	3	7	14	22
Western Gotland Basin	23	18,900	1.22	239	79	*28*	9	4	9	14	33
Gulf of Riga	15	10,400	1.44	121	40	24	*11*	11	30	25	27
Northern Baltic Proper	23	25,275	0.91	308	106	37	*15*	2	5	8	13
Gulf of Finland	28	31,900	0.88	369	111	*36*	14	1	5	14	36
Åland Sea	11	2875	3.83	36	13	*6*	4	22	31	33	50
Bothnian Sea	35	52,400	0.67	551	153	46	*15*	5	14	39	73
The Quark	18	3950	4.56	54	*18*	8	4	19	44	50	50
Bothnian Bay	53	23,550	2.25	260	75	*24*	8	8	20	42	75
Total	298	287,800	3.83	3175	980	346	*137*	5	13	23	36

#### Variance components

For both BQI versions, the variability among stations (${s}_{ST}^2$) is consistently the most dominant component in all models and the interactive variability (${s}_{YR\times ST}^2$) measuring the “inconsistency of year to year variability among stations” is consistently the second most important component (Table 4). Another substantial source of variability is that among replicate samples within a station and year (${s}_{RES}^2$). Finally, we can also note that the overall variability among years within a 6-year assessment period (${s}_{YR}^2$) is quite small.

**Table 4:** Estimated variance components for BQI. BQI1 represents the traditional formulation of BQI (Leonardsson et al. 2009) used in the northern sub-basins and BQI2 represent that used in the southern Baltic (Schiele et al. 2016). In Eq. 2, the variance stemming from broad habitat types (*bh*) in incorporated into the station component (*ST*). See text for further explanation of the variance components

Component	BQI1		BQI2	
	Eq. 1	Eq. 2	Eq. 1	Eq. 2
*YR* × *ST(bh, sb)*	1.45	-	0.31	-
*YR* × *ST(sb)*	-	1.46	-	0.32
*ST(bh, sb)*	1.87	-	1.12	-
*ST(sb)*	-	2.41	-	1.30
*YR*	0.11	0.12	0.01	0.01
*RES*	0.56	0.56	0.30	0.30

For both formulations of BQI, estimates of all components, except that among stations, are not affected by whether the broad habitat types (*bh*) are included in the model. This only matters for the estimation of ${s}_{ST}^2$. As predicted, the variability among stations increases if habitats are not accounted for (BQI1: + 29%, BQI2: + 16%).

#### Precision of BQI

**Fig. 3:**
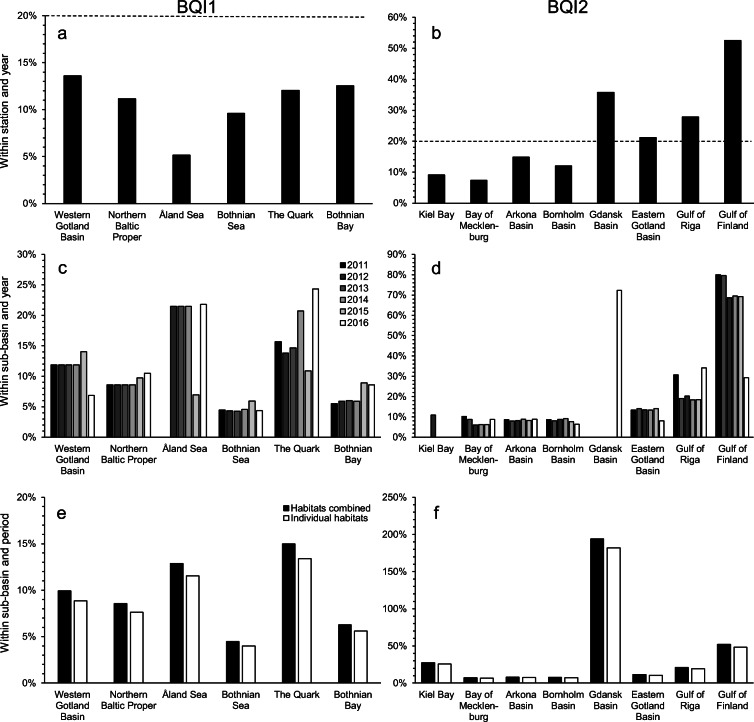
Coefficient of variation (SE/mean) expressed in percentages for BQI1 (**a**, **c**, and **e**) and BQI2 (**b**, **d**, and **f**) for means at spatial and temporal scales of stations within years (**a** and **b**), sub-basins within years (**c** and **d**) and sub-basins within MSFD-assessment periods (**e** and **f**). The striped line at 20% in **a** and **b** correspond to the recommended precision in the monitoring manual (HELCOM 2017)

The precision of the monitoring data varied among sub-basins (Fig. 3). The uncertainty of mean estimates within individual stations and years depends on the number of replicates per station and the variability within stations (see Eq. 6). In general, the standard error at this scale varied 0.3–0.7 BQI units and the smallest errors were found in Gdansk Basin, Bay of Mecklenburg, Åland Sea, and the Quark where a number of replicate samples generally were larger. The patterns were, however, different when put in relation to the mean BQI. For BQI1, all sub-basins had an error smaller than or equal to 20% of the mean (Fig. 3a), which was true also for several sub-basins where the BQI2 was used (Fig. 3b).

The uncertainty of means estimated within sub-basins and years was generally slightly larger than those calculated within 6-year periods (Fig. 3c–f), even though the uncertainty within years contained fewer components of variability. Again, sub-basins assessed by BQI1 had larger SE than those using BQI2, but the difference evened out when uncertainties were estimated relative to the mean. The relative errors were generally in the range of 5–20% of the mean (Fig. 3c–d). Exceptions were the Gdansk Basin and Gulf of Finland where the SE was ≈ 70% of the mean. As expected, the number of stations was mainly determining the differences among sub-basins.

At the scale relevant for status assessment (within sub-basins and 6-year period), the uncertainty of estimated means varied between sub-basins. In the areas where BQI1 was used, the standard error was largest in the Åland Sea, where SE ≈ 1 BQI unit, and the relatively small number of samples appeared to be causing this. In the other sub-basins assessed by BQI1, SE varied between 0.28 and 0.58 BQI and the relative errors were 5–15% of the mean, being highest in the Quark and the Åland Sea (Fig. 3e). In the southern sub-basins, where BQI2 was used, the assessments in Kiel Bay and Gdansk Bay were uncertain with standard errors of ≈ 2 BQI units.^1^ The latter was largely explained by the small number of stations available in this area. Other sub-basins using BQI2 showed standard errors of ≈ 0.3 BQI units. When uncertainties were measured as a proportion of the mean, the situation improved somewhat in Kiel Bay, but in the Gdansk Bay and Gulf of Finland, the precision was still relatively poor (Fig. 3f). Other sub-basins exhibited relative errors of ≈ 10% of the mean. Overall, it was observed that the effects of removing variability due to habitats had a relatively small effect on precision (Fig. 3e, f). In sub-basins where BQI1 was used, the error increased by 12%, whereas is sub-basins where BQI2 was used, an increase by 6–8% was seen when incorporating the variation due to habitat in the station component.

#### Scenario modelling

**Fig. 4:**
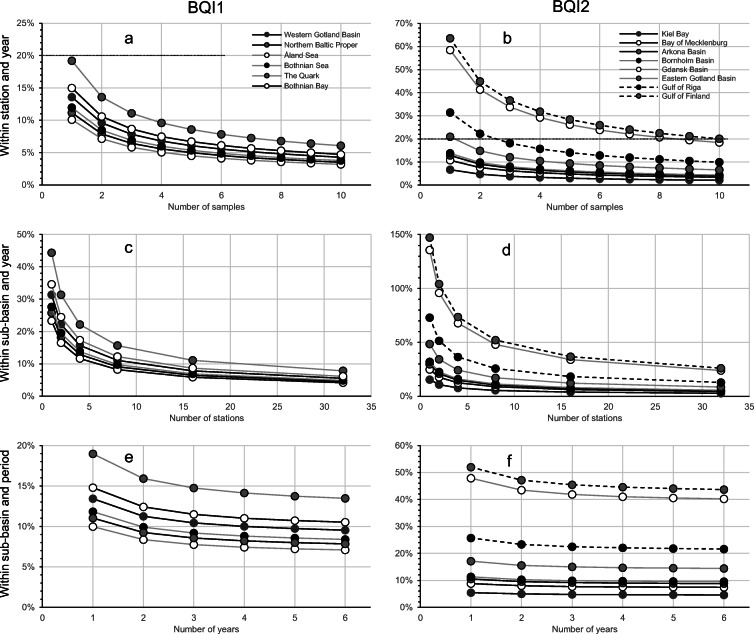
Expected relative uncertainty (SE/mean) expressed in percentages for BQI1 (**a**, **c**, and **e**) and BQI2 (**b**, **d**, and **f**) under varying monitoring scenarios for spatial and temporal scales of stations within years (**a** and **b**; number of samples = 1–10) and sub-basins within years (**c** and **d**; number of samples = 1, number of stations = 1–32) and sub-basins within MSFD-assessment periods (**e** and **f**, number of samples = 1, number of stations = 10, number of years = 1–6). The dotted line at 20% in **a** and **b** correspond to the recommended precision in the monitoring manual (HELCOM 2017)

Modelling the precision within stations and years showed that the estimated variability differed between sub-basins and between the two versions of BQI, but relative to the mean, the precision in most sub-basins met the recommended 20% of the mean at 3–5 samples for both BQI1 and BQI2 (Fig. 4a, b). In several sub-basins, BQI could be estimated at individual stations with reasonable precision even with one replicate sample. Two exceptions were the Gulf of Finland and the Gdansk Basin, likely as a consequence of the low mean BQI values.

The precision within a sub-basin also incorporates variability among stations. Modelling precision at sub-basin level using a different number of replicate samples per station showed that the difference in precision between one and ten replicate samples was < 10%, i.e. the effect of a varying number of replicates was practically negligible in relation to the number of stations and results are thus only presented for models using one replicate. At approximately ten stations per sub-basin the standard error had decreased to a third and the increase in precision levelled off. The recommended precision of 20% of the mean was also achieved in most sub-basins at ten stations, with Gulf of Finland and Gdansk Basin again as exceptions (Fig. 4c, d).

At the most important scale for status assessment, 6-year periods within sub-basins, the precision depends on the variability and efforts in terms of number of samples, stations, and years sampled. As the previous analyses showed that the number of samples per station did not matter in practice, only designs involving *n* = 1 were evaluated (if *n* > 1 SE would be marginally smaller). The error relative to the mean assessment periods was not substantially larger than that among years at comparable number of stations (Fig. 4e, f cf. Fig. 4c, d). Furthermore, the impact of sampling several years was relatively small. For BQI1 and BQI2, the difference between sampling 1 and 6 years is ≈ 35 and 20%, respectively, but note that half of this difference is achieved by sampling 2 years instead of only one (Fig. 4e, f).

**Fig. 5:**
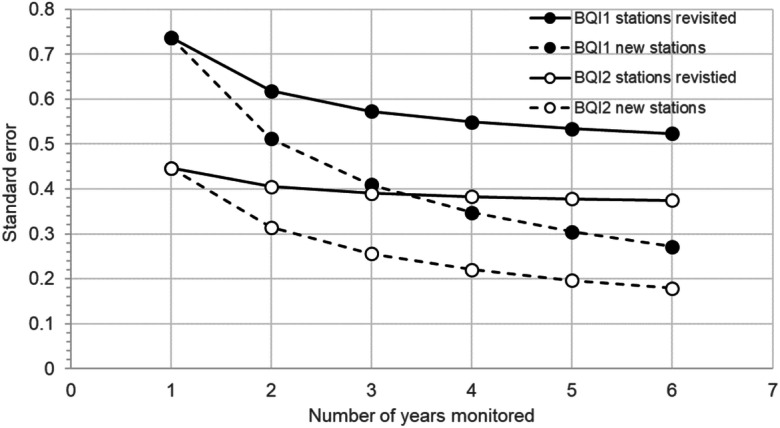
Precision (SE) within assessment period as a function of sampled years for scenarios using “crossed” design revisiting same stations and “nested” design, where new stations are visited each year (number of sampled = 1, number of stations = 10, number of years = 1–6)

The previous analyses were based on a “crossed” sampling design, where the same sampling stations are revisited each year. In contrast, when applying a “nested” sampling design, i.e. choosing new sampling stations each year, the number of years had a considerable impact on precision. Adding new stations (information) each year, the standard error decreased by ≈ 60% between sampling 1 year compared to 6 years (Fig. 5).

## Discussion

This study showed that the current open-sea benthic monitoring programmes in the Baltic Sea, primarily designed to follow up long-term trends in infauna communities, are not the most optimal to use for regional periodic status assessments as defined by the MSFD. Potentially, the same sampling effort could be distributed differently in order to achieve better spatial coverage and more precise estimates on environmental status. Ideally, when designing a monitoring program, a clear purpose of monitoring should be defined in advance (Lindenmayer and Likens 2010). However, in practice, the collected data is often used for several different purposes and these purposes may change over time while the monitoring programme remains the same. Consequently, the big challenge is to evaluate how relevant the data is for the actual purpose. A further challenge in trans-national regional assessments is that countries might have different traditions, priorities, or resources available for monitoring (Van Hoey et al. 2019). This was also reflected in this study, where the review of current monitoring programmes revealed different monitoring practices between the Baltic Sea bordering countries. Whereas all countries monitored soft-bottoms, the sampling methodology and sampling design varied, although common guidelines (HELCOM 2017) are available. Thus, improved cooperation between countries is fundamental to develop the monitoring to allow for more precise regional status assessments.

### Representativity of current monitoring

Benthic habitats are monitored in the open-sea areas of all sub-basins in the Baltic Sea. Although the sampling effort provides reasonable coverage of the dominating habitat types in most sub-basins, the current monitoring is not sufficient to assess all broad habitat types at a regional scale as specified by the MSFD. Current assessments are limited to soft-bottoms, i.e. substrates that can be sampled with grabs, on which the monitoring traditionally has been focused. Broad habitat type maps indicate that current monitoring is covering mud, sand, and mixed sediments, which in fact the dominating substrate types adding up to 94% of the open-sea area. However, monitoring only the dominating habitat types might not be sufficient to assess the region’s biodiversity. Rare and patchy habitat types can form islands of biodiversity hotspots (e.g. Beisiegel et al. 2019). Thus, all habitats need to be considered when allocating resources for monitoring.

In the indicator assessment for HOLAS II, the confidence of the State of the soft-bottom macrofauna community indicator was qualitatively assessed on a three-level scale (high, intermediate, low), based on spatial and temporal representativity of sampling stations (HELCOM 2018c). In addition, the confidence of classification was assessed based on the probability of the indicator result to show the correct status class (GES/sub-GES). Spatial representativity varied between sub-basin from high to low. The temporal coverage was in all sub-basins assessed to be intermediate, as the number of sampling stations visited every year was low. The methods used here can facilitate the estimation of confidence in a more quantitative way.

The issue of spatial and temporal representativity is essential not only from a perspective of its effect on modelled precision in indicator results. The HELCOM open-sea assessment units are large and conditions can potentially be highly variable within the assessment unit. Assessment of status from data representing only a limited part (say north-western) of a sub-basin clearly cannot guarantee that the same thing is happening in another part (i.e. the south-east part) of the same basin. In, e.g. Northern Baltic Proper, Eastern Gotland Basin, and Western Gotland Basin station, density is high in certain areas but very low in other areas, which is translated into a poor coverage in the grid-based approach. Similarly, the status during the first year cannot automatically be assumed in the sixth year. Therefore, it is also important to secure temporal and spatial representativity within sub-basins. The method demonstrated here can provide a useful approach to achieve this. The latter does not mean that all spatial and temporal units must be assessed, but to avoid unnecessary doubts, stations must not be severely geographically biased.

### Implications of monitoring design on precision

The precision of monitoring data used for assessing the benthic macrofauna community in HOLAS II appears to achieve the relative error of 20% benchmark set in this study in most sub-basins at the different scales inspected. The reason for not achieving the benchmark seemed to vary depending on the inspected scales. At the scales “between years within sub-basin” and “within assessment period and sub-basin”, the exceptions seemed to be explained by the low number of stations, whereas for “within stations and years”, it was not the number of samples, but the low mean BQI values in the respective sub-basins that affected the outcome. In the indicator assessment for HOLAS II, the confidence of the State of the soft-bottom macrofauna community indicator was qualitatively assessed on a three-level scale (high, intermediate, low), based on visual inspection of spatial and temporal representativity of sampling stations (HELCOM 2018c). In addition, the confidence of classification was assessed based on the probability of the indicator result to show the correct status class (GES/sub-GES). Spatial representativity varied between sub-basin from high to low. The temporal coverage was in all sub-basins assessed to be intermediate, as the number of sampling stations visited every year was low. The methods used here can facilitate the estimation of confidence in a quantitative way, although the acceptable uncertainty need to be defined.

Based on the variance component estimates, there are persistent spatial differences among stations, clearly seen from the large ${s}_{ST}^2$ variance component, but also some differences in the way the stations change among years. The small overall variability among years within a 6-year period does not mean that there are no changes among years, but that changes differ among stations within sub-basins as indicated by the ${s}_{YR\times ST}^2$ component. The variance components were found to generally be smaller for BQI2. Whether there are any ecological reasons for this is not clear, but one possible technical reason is that the mean values of BQI2 tends to be smaller than for BQI1 as a consequence of the method used to define species sensitivity values. This also means that the relative variability (i.e. in relation to the mean) may be similar for both indices. The latter appears to be true for the spatial components, while the BQI1 seems to be a little more variable concerning the temporal components. Nevertheless, it is also important to realise that the estimated variance components and means are based on a design which is not entirely representative and, in all respects, ideal. For example, we know that habitat maps are not fully reliable, stations are sometimes clustered and not representative, and samples are not distributed among the stations in a balanced way. Given the precautions taken in interpretation (i.e. primarily applying Eq. 2) and the size of the dataset, however, the conclusions about variability and uncertainty can be expected to be robust. Possibly, some caution should be reserved for the relative estimates of uncertainty due to the strong dependence on the mean, which is sometimes estimated from a small number of stations.

Following the estimates of the variance components, the analyses of precision of monitoring scenarios clearly demonstrate the overriding importance of spatial coverage and of maximising the number of sampling stations in order to achieve reliable and precise status assessments for the Baltic Sea sub-basins. This can be achieved either by increasing the available funds, by reallocating efforts, by reducing the number of samples per station, or by implementing a nested or partly nested design allowing more stations to be sampled with the same resources. The most economically attractive or practical solution to this problem is likely to differentiate among sub-basins and countries, but judging from the quantitative analyses, the path to a more precise monitoring programme and more confident assessments goes through increasing the number of stations. Although the current monitoring programmes deliver data allowing adequate precision in assessments, the same number of samples can provide even higher precision if distributed among more stations. If increasing the number of stations, the geographic coverage of stations should be considered, to eventually increase the spatial representativity of the monitoring. Further investments in monitoring to increase precision in status assessment can prove worthwhile, compared to making wrong management decisions based on vague information (Nygård et al. 2016).

For some purposes, the “crossed” sampling design may be a fundamental requirement and preferred option (Van der Meer 1997), but the benefits of sampling multiple years for an improved precision in regional status assessments are limited. In less technical terms, it can be said that little new information is added during the repeated visits, and that once the station is visited, it is pretty clear how it will look the next year. The analysis shows that in contrast to the “crossed” design, the number of years has a significant impact on precision with a “nested” sampling design. Because new stations (information) are added each year, the error decreases by ≈ 60% between sampling 1 year compared to 6 years. Although such a sampling design probably is a little bit more costly due to the process of finding new stations, the total sampling effort in terms of samples and stations is kept constant (e.g. 6 years × 10 stations × 1 sample = 60 samples) while the number of unique stations would be ten with the crossed design and sixty stations with a fully nested design. It may also be possible to find useful compromises between these two approaches. In fact, such combined approaches are at least partly implemented in, for example, the Swedish zoobenthic monitoring programme (Leonardsson and Blomqvist 2015), where some stations are visited at even years and others at odd years. The latter approach has now been partially implemented in all Swedish coastal seas and is expected to lead to significantly increased precision in status assessment, a more complete and representative estimate in more water bodies (sensu WFD), while still maintaining opportunities for time series analyses at individual stations.

Even though the BQI has proven useful in assessing impacts of a variety of pressures (e.g. Josefson et al. 2009; Chuševė et al. 2016; Gislason et al. 2017), formulation of the index and selection of species sensitivity values may affect the outcome (Fleischer et al. 2007; Fleischer and Zettler 2009; Leonardsson et al. 2015; Chuševė and Daunys 2017; Gislason et al. 2017). The analyses of precision done here are focused on the HELCOM core indicator (BQI) and are not automatically applicable to other indicators or metrics of the status of the benthic community or individual taxa. Furthermore, whether the different broad habitat types can be assessed using the same approach (indicator, threshold) is not clear, as reference conditions can vary depending on habitat type (Van Hoey et al. 2013). Nevertheless, the holistic status assessments are fundamental for evaluating regional policies in the Baltic Sea and for complying with European laws. Therefore, monitoring programmes need to be thoroughly evaluated and adapted so that precision and confidence are optimised, or at least known, with respect to meeting these important policies.

Finally, it is worth noting that it is not primarily the sampling effort (number of samples and number of stations) that limit the ability to achieve full coverage of most sub-basins with a higher resolution than today, and our results imply that the current monitoring programmes have potential for improvements at unchanged cost. Of course, there may be many rational or historical reasons for why the current monitoring programmes or parts of the programmes are designed as they are. Clearly, the analysed monitoring stations serve other purposes than the status assessment of sub-basins according to the HOLAS or MSFD process; for example, continuation of time series data of biodiversity, particularly important taxa or functional groups at individual stations or in national territories. Sustaining long-term time series can, besides improving the understanding on natural variability, prove valuable in studying effects of slowly emerging changes, e.g. climate change (Zettler et al. 2017; Kuosa et al. 2017). Time series are also pivotal in identifying thresholds for status indicators, as the time series often span over time scales with differing levels of human impact (Villnäs and Norkko 2011). Consequently, a broad-brushed reallocation of the resources is not recommended. Nevertheless, it is also possible that lack of quantitative analyses; benefits of alternative strategies; and less rational, historical reasons as well as insufficient coordination among countries are limiting potential revisions towards more effective monitoring designs. To reach a more optimal spatial distribution of monitoring stations in the open-sea areas, covering all habitat types, improved cooperation between countries is needed, although it might compromise the national preferences. Cooperative efforts are also needed to streamline methodology and in developing common monitoring guidelines.

## Conclusions

The review of current monitoring programmes highlights that benthic habitats are unevenly monitored in the open-sea areas of the Baltic Sea and the geographic representativity of sampling stations is unbalanced. Whereas soft-bottoms have been monitored > 50 years, monitoring of other habitat types have only recently started in parts of the Baltic Sea, restricting the possibilities to perform regional assessments of all MSFD broad habitat types. Although the monitoring data provide reasonable confidence in assessments of soft-bottom macrofauna communities, the analyses show that both precision and representativity of the data can be improved keeping the sampling effort constant, but re-designing the monitoring programmes. Redistributing stations can improve representativity of the sub-basins, whereas redistributing samples among an increased number of stations is the most advantageous action in terms of increasing precision in assessments. However, changes to the monitoring programmes need to be considered carefully in order to not compromise the valuable long-term data series available in the Baltic Sea. When developing the monitoring of benthic habitats in the Baltic Sea, it is recommended that cooperation between the bordering countries is increased, to overcome challenges arising from differing priorities, methodology, or resources.

## Data Availability

Information collected on the monitoring programmes is presented in the paper. The data used for analyses of precision of the “State of the soft-bottom macrofauna community” is openly available from the HELCOM Map and Data Service (http://metadata.helcom.fi/geonetwork/srv/eng/catalog.search#/metadata/0fdc6cb9-fa15-4ba3-9d73-8aeddb5cff64).

## Footnotes

1 Note that this estimate involves only available monitoring data in Kiel Bay and not all data that was used for HOLAS II.
